# Maintained gait in persons with arthrogryposis from childhood to adulthood

**DOI:** 10.1186/s12891-025-08366-9

**Published:** 2025-02-12

**Authors:** Åsa Bartonek, Mikael Reimeringer, Marie Eriksson

**Affiliations:** 1 Department of Women’s and Children’s Health, Karolinska Institutet, Stockholm, Sweden; 2 Karolinska University Hospital, Stockholm, Sweden; 3 Astrid Lindgren’s Children’s Hospital, Karolinska University Hospital, Karolinska Vägen 37A, Stockholm, 17176 Sweden

**Keywords:** Ambulation, Gait analysis, AMC, Orthoses, Adulthood, Satisfaction

## Abstract

**Background:**

Individuals with arthrogryposis multiplex congenita (AMC) exhibit a range of modes of ambulation, from walking independently to requiring a wheelchair. Presence of joint contractures and muscle strength plays a crucial role, and, in some patients, orthoses are necessary to facilitate or enable walking.

**Methods:**

Gait was assessed with a three-dimensional (3D) gait analysis, calculated as a gait deviation index (GDI) of nine kinematic variables, and compared between childhood and adulthood.

**Results:**

A total of 12 persons, 8 with community and 4 with household ambulation, who had undergone a 3D gait analysis in childhood (CH) and as an adult (follow-up, FU) at the same gait laboratory were enrolled in the study. At the FU, three, five, and four participants respectively were categorized based on need of joint stabilization while walking as AMC1 using knee-ankle-orthoses (KAFOs) with locked knee joints, AMC2 using KAFOs with free-articulating knee joint or ankle–foot-orthoses (AFOs) and AMC3 using insoles or shoes. Two participants in AMC2 had changed from AFOs to insoles or shoes between CH and FU. There were no differences in joint contractures between the AMC groups at CH or FU. Two participants had orthopaedic surgery between CH and FU. The GDI of the leg with the lowest GDI score at CH vs FU was median [min, max] 55.67 [41.79, 65.14] vs 48.4 [42.67, 56.30] (*p* = 1.000) in AMC1, 81.25 [59.42, 84.12] vs 68.96 [47.68, 70.33] (*p* = 0.043) in AMC2, and 73.15 [43.94, 91.72] vs 73.46 [50.82, 75.24] (*p* = 1.000) in AMC3. Time and distance parameters of cadence, walking speed, step length, and step width did not differ between the CH and FU, nor were there differences in satisfaction with the device or the service at the FU.

**Conclusion:**

A difference in the GDI was found in one of the AMC groups between childhood and adulthood that could not be explained by factors such as contractures or muscle strength. This study reflects that gait is maintained in ambulating persons with AMC who were offered an orthosis program that has been available from childhood into adulthood.

**Supplementary Information:**

The online version contains supplementary material available at 10.1186/s12891-025-08366-9.

## Background

Patients with arthrogryposis multiplex congenita (AMC) are seen regularly by rehabilitation teams, with each specific type of AMC being relatively rare [1]. In the group of AMC, patients exhibit a range of modes of ambulation, from walking independently to requiring a wheelchair. In some patients, orthoses are necessary to facilitate or enable standing or walking. The use of an orthosis and the walking function have been reported in children with AMC through an assessment of kinematics and effort required. The reports show that walking can be achieved even with severe weakness and contractures leading to recommendations of the continuous use of orthoses for persons with AMC [2–5]. Given the extensive level of orthopaedic and orthotic management during childhood, it is of interest to explore the ambulatory level at adulthood.

AMC is defined as the presence of two or more congenital contractures in different areas of the body and may be caused by factors that affect fetal movement, including neuromuscular disease (intrinsic), maternal illness (environmental), medications, or limited in utero space (extrinsic). The contractures are secondary to decreased fetal movement, which can be due to a variety of causes, including nerve-, muscle-, and connective tissue–related pathologies [6]. In Canada, during 1997–2007 the prevalence for multiple congenital contractures has been found to be 1/4300. Specific diagnostic categories such as limbs with and without noncentral nervous system anomalies have been reported and a new classification has been proposed [7].

There are two types of arthrogryposes of which the first type is one of the accompanying signs in the context of various pathologies, particularly neuromuscular diseases. In the second, the arthrogryposis is the main and constant symptom representing two specific forms, one of amyoplasia with significant congenital absence of muscles and distal arthrogryposis which has a genetic component [8]. Amyoplasia is usually characterized by symmetric involvement of the limbs, with extension of the elbows, extension or flexion of the knees, and contractures or dislocation of the hips. In amyoplasia, muscle function affects motor function to a greater extent than contractures and joint position at birth [9]. Knee extension contractures and clubfoot deformities have been associated with dislocated hips with underlying diagnoses of amyoplasia, distal arthrogryposis, and central nervous system types of AMC, whereas bilateral hip dislocations have been rarely seen in individuals with distal arthrogryposis [10]. Also, the central nervous system is considered to be a cause for a lack of movement in utero, emphasising the importance of a correct diagnosis in planning adequate possible new treatments [11].

Contractures are usually non-progressive and improve over time with early physiotherapy and appropriate orthopaedic care, and two-thirds of affected individuals are able to live independent and productive lives [12]. Therapeutic interventions for AMC have not changed much over the past 50 years and include nonsurgical approaches such as splinting for upper extremity contractures and surgical approaches for lower extremity contractures [6]. In a large study on adults with AMC, the orthopaedic surgeon was the primary specialist involved in the participants’ care and nearly 55% of the participants had gone through more than 5 surgeries [12].

In the management of lower limb deformities in children with arthrogryposis (specifically amyoplasia), very early and extensive management of these deformities in the form of intensive physiotherapy and bracing is important [9]. Since approximately the mid-1990s, children with AMC treated in Karolinska University hospital in Stockholm have been offered orthotic management similar to a concept originally developed for children with myelomeningocele (MMC) [13]. In this concept, planning for possible ambulatory ability starts from an early age with adequate orthotic support customised to the needs of each individual. Through a close cooperation of the PT with the orthotic clinic that provided all orthoses, attention is given to achieve correct alignment and adequate material for each orthosis taking into account the aspects of timing of orthoses prescription with respect to the child’s motor development and motivation to move*.*

In adults with MMC the results from a follow-up study show a high frequency of orthosis use in adult age. The findings emphasize that early planning and follow-up of orthosis treatment during growth are important for mobility in adulthood, however, a close assessment of the condition of each patient is very important [14]. Children with AMC have been provided with an active orthotic concept since childhood with largely the same orthosis models as used for persons with MMC at sacral and low and mid lumbar levels. Gait can be expressed in the form of a gait index [GDI] based on kinematic parameters [15]. The aim of this study was to follow up the gait in an adult population with AMC. The hypothesis was that the GDI would remain consistent in persons who have continued to walk as an adult.

## Methods

Of the group of 24 adults with AMC in the Stockholm region in 2018–2019 who were invited to participate in a survey concerning function, orthosis use, and ambulation, 2 declined to participate, 7 did not respond to the invitation of which 3 had moved from the region, thus 15 adults agreed to take part in the survey. Inclusion criteria were that they were born after 1985, and they were older than 18 years. From that group of 15, 5 women and 7 men who had undergone a three-dimensional (3D) gait analysis in childhood (CH) and a follow-up gait analysis (FU) in the current survey as an adult at the same gait laboratory were enrolled in the present study.

Age at CH was mean (standard deviation, SD), 13.8 (2.7) years, with a range of 7–18 years. Age at FU was mean (SD) 24.5 (3.3) years, with a range of 19–29 years. The time between the CH and FU gait analyses was mean (SD) 11.1 (2.1) years, with a range of 6–12 years.

The study was approved by the Regional Ethical Review Board in Stockholm, Dnr. 2017/910–31/4. Informed and written consent was obtained from all participants.

### Orthoses

The orthosis types used by the participants were grouped based on the international classification [16] as foot orthosis (FO), ankle–foot orthosis (AFO), and knee-ankle–foot orthosis (KAFO). A modification was made by specifying the KAFOs as constructed with either a free-knee articulating joint in the sagittal plane or with a locked knee joint. AFOs were distinguished as solid (AFO-S), hinged (AFO-H) [4], or constructed with a carbon fibre spring in the ankle (AFO-C) [17]. Material and components used in the orthoses are shown in Appendix A.

### AMC subgroups based on need of joint stabilization

The participants were divided into subgroups based on their need of joint stabilization with orthoses while walking. AMC1 represented participants who required KAFOs with locked knee joints (KAFO-LK) to support weak knee extensors. In AMC2, AFOs to stabilize the ankle or KAFOs with free-articulating knee joints (KAFO-F) to prevent knee motion in the frontal and transverse planes were used. AMC3 represented those who required non-ankle restricted FOs to support the plantar surface of the foot [4]. In participants with plantarflexion contractures, material was applied on the orthoses on the heel and with heel wedges on the shoe sole for final alignment of the body segments if necessary [3]. Use of orthoses were documented at FU and retrospectively at CH through the records in the certified prosthetic and orthotic (CPO) clinic. All orthoses were delivered by the same orthotic provider.

### Satisfaction with orthoses

Satisfaction with orthoses was evaluated using two of the five modules in the Swedish version of the Orthotics and Prosthetics Users’ Survey (OPUS): client satisfaction with the device (CSD), comprising 9 items, and client satisfaction with services (CSS), comprising 10 items [18].

### Clinical examination and functional ambulation

Joint motion in hip, knee, and ankle was performed in a supine position with a goniometer. A contracture was defined from a neutral joint position. Hip flexion contracture was examined according to the procedure of Bartlett [19].

Muscle strength examination in the lower limb muscles was assessed on a 0–5 graded scale. Grades 4 (“good”) and 5 (“normal”) were defined as the ability to withstand considerable manual resistance when being asked to hold the leg and not allowing the examiner to break the hold. Grade 3 (“fair”) indicated the ability to hold the leg against only the resistance of gravity. Grade 2 (“poor”) was the ability to complete the motion in a position that minimizes the force of gravity. Grade 1 (“trace”) was defined as when some contractile activity was detected by the examiner visually or by palpation. Finally, Grade 0 (“zero”) meant that the muscle was completely quiet on palpation or visual inspection [20]. Participants walking outdoors or indoors were designated as with community ambulation or household ambulation, respectively [21].

### Orthopaedic surgical history

Procedures performed on the lower limbs, pelvis, and spine for each participant were retrieved from the medical records.

### Gait analysis

The participants were tested with a 3D, 8-camera motion analysis system (Vicon MX40, Oxford, UK) with a sampling rate of 100 Hz. A conventional full-body biomechanical model with 34 retroreflective markers attached to anatomical landmarks on the head, trunk, pelvis, and lower limbs (Plug-In-Gait) was used, based on the Newington model [22]. The markers were placed on the orthoses as near as possible, relative to the anatomical joints and body segments. Static calibration of the Plug-In-Gait model was conducted with ‘assume horizontal’ selected to align the foot segment with the floor plane, to be able to measure the heel-toe gait with the orthoses*.*

Data were collected with participants walking at a self-selected velocity on an eight-meter walkway. The trials were performed with each participant’s current orthoses and/or shoes.

### Data and statistical analysis

A minimum of three gait cycles per side were collected for each participant. A Gait Deviation Index (GDI), summarizing nine kinematic parameters: pelvic and hip angles in the frontal, sagittal, and transverse planes, knee flexion/extension, ankle dorsiflexion/plantarflexion, and foot progression into a multivariate measure of gait deviations, was estimated separately for the left and right sides. The calculation of the GDI [15] derives from 6702 barefoot strides from a paediatric population. A GDI of 100 or higher indicate a person whose gait was at least as near to the average of typically developing (TD) subjects as that of a randomly chosen TD person. The GDI sample consists of children with various walking levels from community ambulation to keeping up with peers according to the FAQ (Functional Assessment Questionnaire) parent-report walking scale according to Novacheck et al. 2000 [23]. Except children with cerebral palsy representing the majority, also children with traumatic brain injury, myelomeningocele and even idiopathic toe walking are included in the population [23].

Kinematic parameters of pelvic and hip angles in the frontal, sagittal, and transverse planes, knee flexion/extension, ankle dorsiflexion/plantarflexion, and foot progression were obtained from each of the gait cycles and averaged for each side at the CH and FU gait analyses. The side with the lowest average GDI score was used for illustration of the kinematic variables summarized in the GDI. In addition, trunk angles in the frontal, sagittal, and transverse planes were averaged from each of the three gait cycles of the leg with the lowest GDI. Time and distance parameters of cadence, walking speed, step length, and step width were averaged of three trials for each side.

Statistical analyses were performed using commercially available software (SPSS version 28.0). Descriptive data are presented as mean and standard deviation, median, minimum (min), and maximum (max) values. The differences in GDI for the left and right side with respect to each of the AMC groups were analysed using the Wilcoxon sign rank test and to compare GDI, time and distance parameters and joint contractures between gait analysis in childhood and in adulthood. Time and distance parameters of cadence, walking speed, step length, and step width were non-dimensionalized [24] and presented as a mean of both sides. A Kruskal Wallis test was used to compare joint contractures, GDI and orthosis weight between the AMC groups. The level of significance was set at *p* < 0.05.

Gait analyses results are reported in one participant who had performed gait analysis only at FU but not in childhood.

## Results

At the FU, three participants were in AMC1, five in AMC2, and four in AMC3. Two participants had changed from AMC2 to AMC3 between CH and FU. Eleven of the 12 participants had some upper limb (UL) involvement. Eight participants had community ambulation and four had household ambulation. None of the participants had a change in functional ambulation between CH vs FU, and none used a walking aid.

Table 1 shows AMC subgroups, type of AMC, upper limb involvement, orthoses, functional ambulation, joint contractures defined from a neutral joint position, and muscle strength at the gait analysis at CH and FU.

**Table 1:** AMC subgroup, orthoses, upper limb involvement, functional ambulation, joint contractures and muscle strength at gait analysis in childhood (CH) and at gait analysis at follow-up (FU), with respect to participants in AMC1, AMC2, and AMC3

N	AMC sub-group	AMC type	UL	Orthoses	Amb	Joint contractures						Muscle strength									
				CH/FU		CH	FU	CH	FU	CH	FU	CH	FU	CH	FU	CH	FU	CH	FU	CH	FU
						HE		KF		PF		HF		HE		HA		KE		PF	
						L/R	L/R	L/R	L/R	L/R	L/R	L/R	L/R	L/R	L/R	L/R	L/R	L/R	L/R	L/R	L/R
1	1/1	A	W E S	KAFO-KL-C/KAFO-KL-C	Ha/Ha	-	**−5/**	**−15/**	**−15/**	**−20/−20**	**−20/−20**	3/3	3/4	4/4	4/3	4/4	4/5	3/3	2/4	0/0	0/0
2	1/1	U	H W	KAFO-KL/KAFO-KL	Ha/Ha	-	**−5/**	**−30/−25**	**−30/−25**	-	/**−10**	4/4	5/5	4/4	5/5	3/4	4/4	4/4	4/4	0/0	2/4
3	1/1	A	H W E S	KAFO-KL-C/KAFO-KL-C	Ha/Ha	-	-	**−30/−25**	**−10/−15**	**−20/−15**	**−30/−40**	3/3	4/4	4/4	5/5	4/4	5/5	3/3	3/3	1/1	0/0
4	2/2	D	H W E S	AFO-S/AFO-C	Ca/Ca	-	-	-	-	**−20/0**	-	4/4	5/5	4/4	5/5	4/4	5/5	4/4	5/5	4/4	0/0
5	2/2	U	-	AFO-C/AFO-C	Ca/Ca	-	**/−5**	**−10/−10**	**-**	**−20/−20**	-	4/4	5/5	4/4	5/5	4/4	5/5	4/4	5/5	0/0	0/0
6	2/2	A	W E S	KAFO-F–C L & AFO-C R/KAFO-F–C L & AFO-C R	Ha/Ha	-	-	-	-	-	-	4/4	4/4	4/4	4/4	4/4	4/4	4/4	4/4	2/2	0/0
7	2/3	A	W E S	AFO-H/FO-insole	Ca/Ca	-	-	**−10/−5**	**−5/**	-	**−10/**	4/4	4/4	4/4	5/5	4/4	5/5	4/4	5/5	0/3	2/4
8	2/3	P	H W E S	AFO-S L/Shoe	Ca/Ca	-	-	**/−5**	**−5/−10**	-	-	4/4	5/5	4/4	4/4	4/4	5/5	5/5	5/5	5/3	5/4
9	3/3	U	E	FO-insole/FO-insole	Ca/Ca	**−20/−15**	**−25/−25**	-	-	**−15/−15**	**−10/**	4/4	4/4	4/4	5/5	4/4	4/4	4/4	5/5	4/4	4/5
10	3/3	D	E S	FO-Insole/FO-insole	Ca/Ca	-	-	-	-	-	-	4/4	4/4	4/4	5/5	4/4	5/5	4/4	5/5	4/4	5/5
11	3/3	U	W E	Shoe/Shoe	Ca/Ca	-	/**−5**	-	-	-	-	5/5	5/5	5/5	5/5	5/5	5/5	4/4	5/5	4/4	5/5
12	3/3	U	E	Shoe/Shoe	Ca/Ca	-	-	-	-	-	-	4/4	5/5	4/4	5/5	4/4	5/5	4/4	5/5	4/4	5/5

*A* Amyoplasia, *U* Unspecified type, *D* Distal arthrogryposis, *P* Pterygium syndrome, *UL* Upper limb involvement, *H* Hand, *W* Wrist, *E* Elbow, *S* Shoulder, *HE* Hip extension, *KF* Knee flexion, *PF* Plantarflexion, *HF* Hip flexion, *HA* Hip abduction, *KE* knee extension, *PF* Plantar flexion, *Amb* functional ambulation, *KAFO* Knee-ankle–foot orthosis, *KL* locked knee joint, *C* Carbon fibre spring, *F* Free-articulated knee joint, *FO* Foot orthosis, *AFO* Ankle–foot orthosis, *S* Solid, *H* Hinged with restricted range of motion, *bi* bilateral, *L* left, *R* right, *Ca* community ambulation, *Ha* household ambulation

### Orthoses

At CH, 10 of 12 participants wore orthoses and the other 2 wore shoes. At FU, one participant in AMC2 had changed from AFOs to FOs and one from AFOs to shoes. At CH and FU, four and five participants, respectively, wore AFO-Cs (14). The types of orthoses used by the participants are shown in Table 1. Orthoses and material are illustrated in Appendix A.

Of the 9 participants wearing orthoses at FU, eight used their orthoses daily and one used the orthosis > 4 days/week. On the days they wore orthoses, 5 participants wore them > 10 h/day and the other 4 wore them > 8 h/day.

The weight of both orthoses in AMC1, AMC2, and AMC3 were median [min, max] 4.5 [4.3, 5.1], 2.0 [0.5, 2.4], and 0.4 [0.4, 0.8] kilogrammes, respectively (*p* = 0.014). A post hoc test revealed a higher orthosis weight in AMC1 compared to AMC2 (*p* = 0.036).

### Joint contractures and muscle strength

Joint flexion contractures as measured from a neutral joint position in the sagittal plane are shown for left and right legs at CH and FU in Table 1. There were no differences in hip flexion contractures either in the left or right leg, nor in knee flexion contractures left (*p* = 0.180), and right (*p* = 0.655), or in plantar flexion contractures left (*p* = 0.655) or right (*p* = 0.317). There were no significant differences in joint contractures between the AMC groups in any joint at CH and FU.

At both CH and FU, most participants had grade 4 to 5 in muscle strength in hip flexion, hip extension and hip abduction. At CH, hip flexion was assessed as a grade 3 in five legs of which four had changed to a grade 4 at FU. One participant had a grade 3 in hip extension at FU, and one a grade 3 at CH in hip abduction. Plantarflexion among the participants ranged between grade 0 and grade 5 at both CH and FU.

### Orthopaedic surgical history

All participants had gone through orthopaedic surgery. Procedures performed before gait analysis in childhood and between CH and FU are shown in Table 2.

**Table 2:** Orthopaedic surgical procedures performed before gait analysis in childhood (CH) and between CH and follow-up gait analysis (FU)

Subject	Orthopedic surgery	
	Before CH	Between CH and FU
1	1y: Club foot bi 7y: Pelvic osteotomy R 11y: Pelvic osteotomy L	
2	> 1y: Club foot bi 6y: Hamstrings lengthening bi, femoral derotation bi 11y: Subtalar arthrodesis bi, tibialis anterior transfer L	
3	< 1y: Club foot bi 5y: Hamstrings lengthening bi 15y: Flexor hallucis longus lengthening R, plantar capsulotomy R	
4	< 1y: Club foot bi 9y: Ilizarow foot correction bi 12y: Correction osteotomy foot and arthrodesis R, Correction osteotomy foot L 17y: Correction osteotomy foot, resection dig III and IV L 21y: resection dig II-IV R	
5	1y: Femoral osteotomy L 13y: Correction osteotomy foot L	
6	6y: Pelvic osteotomy, femoral derotation and varus osteotomy, Club foot bi	22y: Total hip replacement L
7	1y: Achilles tenotomy bi 2y: Club foot bi 3y: Plantar fasciotomy, Abductor hallucis longus lengthening, cuboid osteotomy	
8	7y: Insertion of VEPTR for correction of scoliosis 10y: untethering spinal cord, Spine fusion, Akilles lengthening L, dorsal capsulotomy L	
9	2y: Open reduction hip bi, Club foot bi 10y: Club foot L, Ankle osteotomy L 15y: Redression of knee dislocation R	
10	1y: proximal femoral derotation and varus osteotomy bi, Club foot L, Adductor tenotomy bi 12y: Femural epifyseodesis R	
11	12y: Guided growth distal femur bi, Akilles lengthening R, dorsal capsulotomy R	
12	1y: Pelvic osteotomy R, proximal femur osteotomy L, Femur distal fyseodesis R	18y: Quadriceps lengthening R, collateral ligament lengthening

*Bi* bilaterally, *y* years, *R* right, *L* left, *VEPTR* vertical expandable prosthetic titanium rib

### Gait deviation index (GDI)

There were differences in the left and right sides of GDI between the CH and FU in AMC2. The left side GDI in AMC2 was median [min, max] 84.1 [59.4, 87.4] vs 69.7 [47.7, 82.9] (*p* = 0.043), and the right side GDI was median [min, max] 81.3 [60.2, 84.7] vs 70.9 [56.8, 74.3] (*p* = 0.043).

**Fig. 1:**
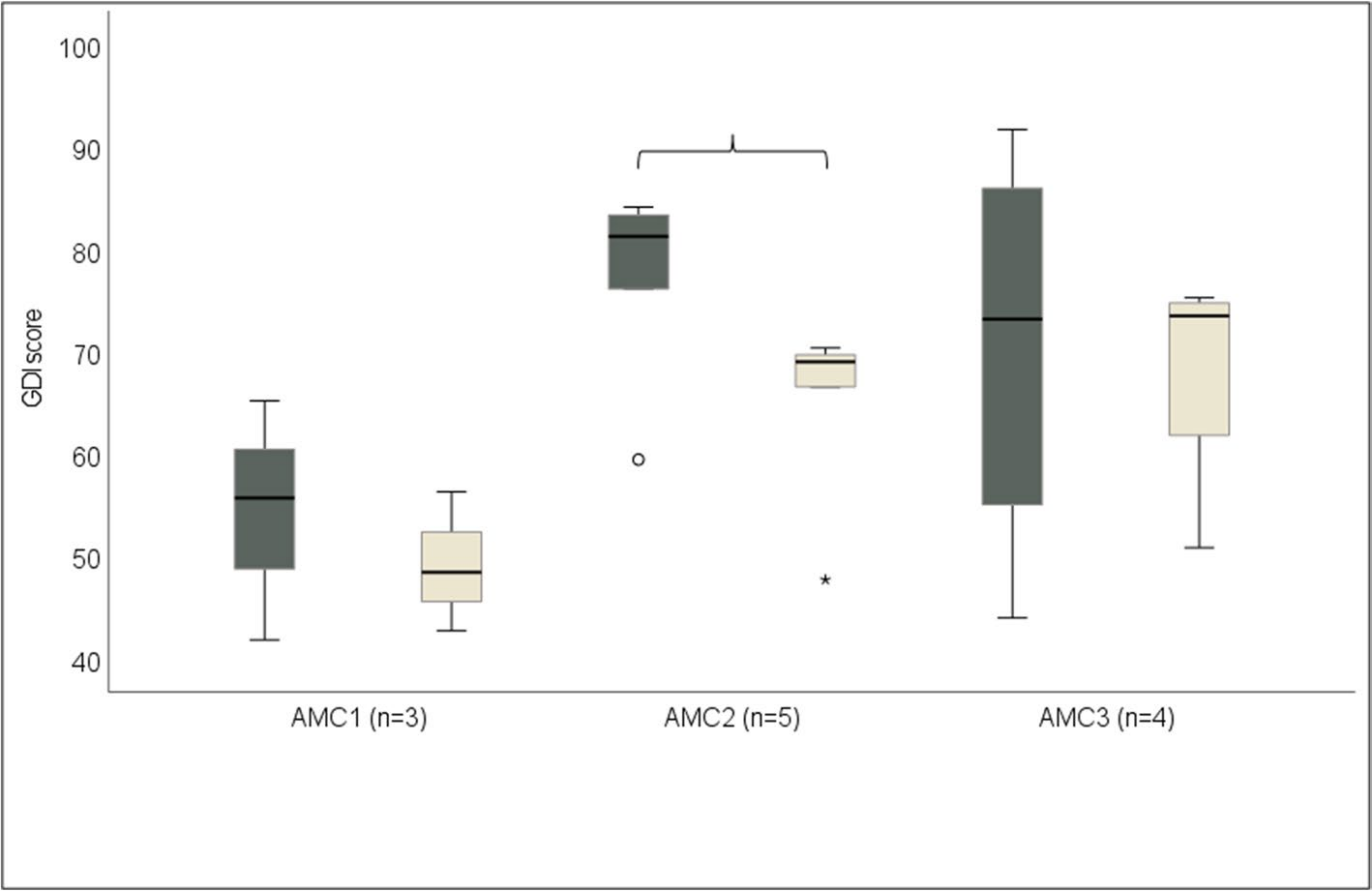
GDI (Gait deviation index) of the leg with the lowest GDI score at CH (childhood gait analysis) (dark grey) and at FU (follow-up gait analysis in adulthood) (light grey) in AMC1, AMC2, and AMC3. Parenthesis indicates statistical difference

The leg with the lowest GDI at CH vs FU in AMC1, AMC2, and AMC3 was median [min, max] 55.7 [41.8, 65.1] vs 48.4 [42.7, 56.3] (*p* = 1.000), 81.3 [59.4, 84.1] vs 69.0 [47.9, 70.3] (*p* = 0.043), and 73.2 [43.9, 91.7] vs 73.5 [50.8, 75.2] (*p* = 1.000), respectively (Fig. 1).

There were no statistical differences between the AMC groups in terms of their average GDI of the leg with the lowest GDI at CH (*p* = 0.179) nor at FU (*p* = 0.076).

### Illustration of gait

**Fig. 2:**
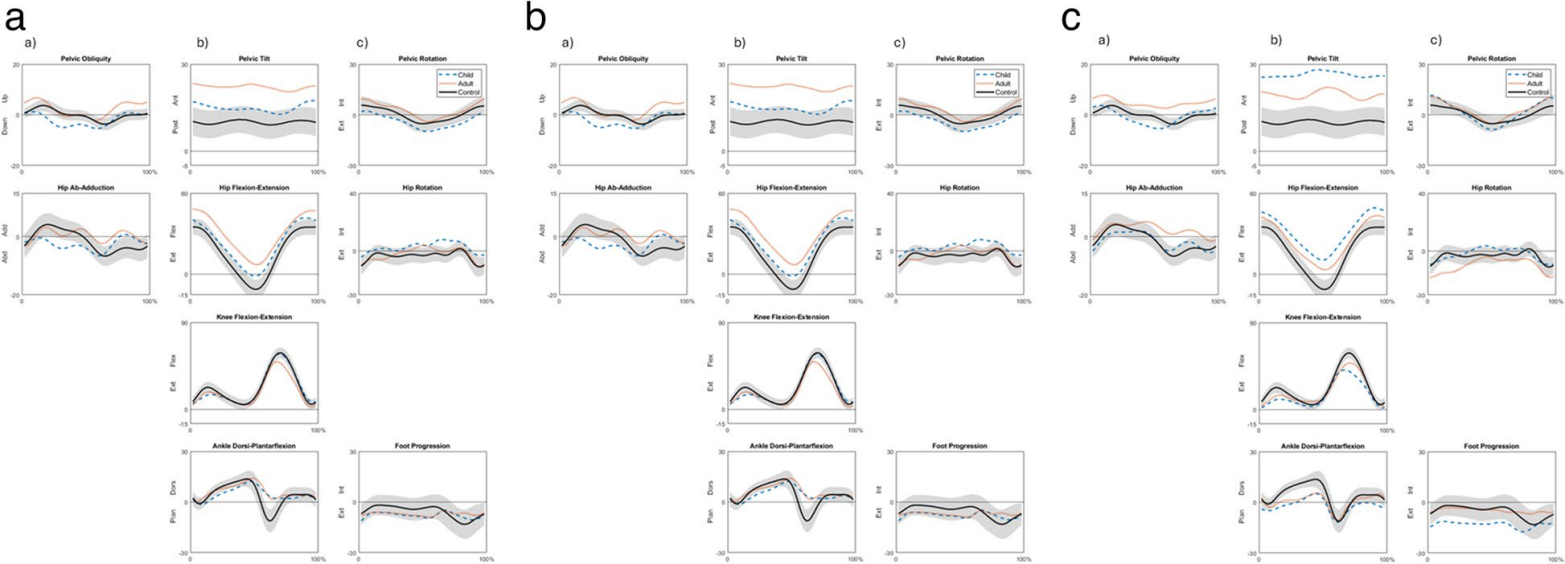
a. Illustration of the nine parameters that are summarized in the GDI in the a) frontal, b) sagittal, and c) transverse planes (pelvic and hip angles, knee flexion/extension, ankle dorsiflexion/plantarflexion, foot progression) as the mean of three trials of the leg with lowest GDI at CH (Child) and FU (Adult) in AMC1. The shaded field represents the mean ± 1 standard deviation of 37 control children of the same gait laboratory (Control). b. Illustration of the nine parameters that are summarized in the GDI in the a) frontal, b) sagittal, and c) transverse planes (pelvic and hip angles, knee flexion/extension, ankle dorsiflexion/plantarflexion, foot progression) as the mean of three trials of the leg with lowest GDI at CH (Child) and FU (Adult) in AMC2. The shaded field represents the mean ± 1 standard deviation of 37 control children of the same gait laboratory (Control). c. Illustration of the nine parameters that are summarized in the GDI in the a) frontal, b) sagittal, and c) transverse planes (pelvic and hip angles, knee flexion/extension, ankle dorsiflexion/plantarflexion, foot progression) as the mean of three trials of the leg with lowest GDI at CH (Child) and FU (Adult) in AMC3. The shaded field represents the mean ± 1 standard deviation of 37 control children of the same gait laboratory (Control)

Gait kinematics at CH and FU are illustrated through the nine parameters which are summarized in the GDI (i.e., pelvic and hip angles in the frontal, sagittal, and transverse planes, knee flexion/extension, ankle dorsiflexion/plantarflexion, foot progression) during the whole gait cycle as the mean of three trials of the leg with lowest GDI at CH and FU (Fig. 2a–c).

**Fig. 3:**
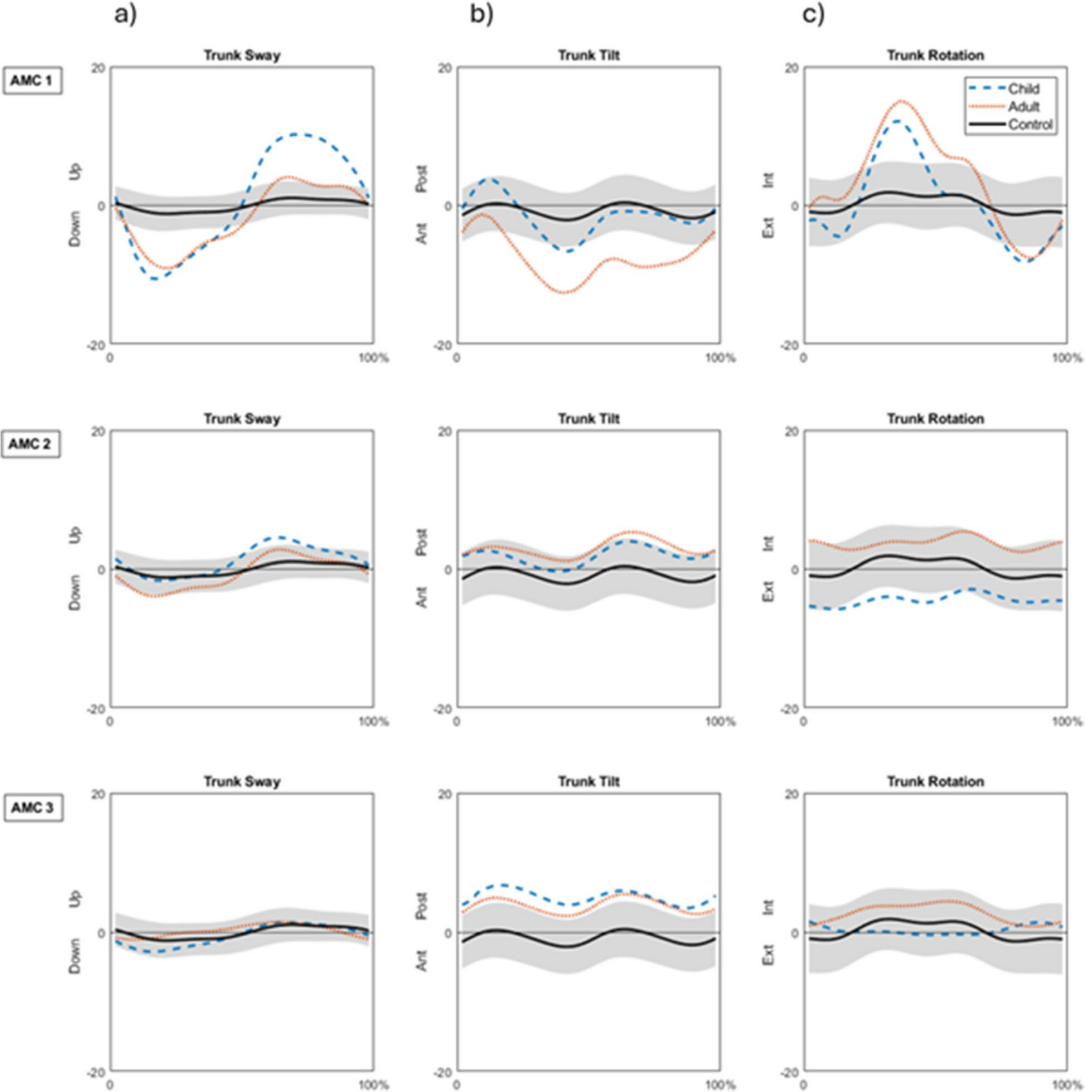
Illustration of trunk movements in the **a**) frontal, **b**) sagittal, and **c**) transverse planes during the whole gait cycle as the mean of three trials of the leg with lowest GDI at CH (Child) and FU (Adult) in the AMC1, AMC2, and AMC3 groups. The shaded field represents the mean ± 1 standard deviation of 37 control children of the same gait laboratory (Control)

Illustration of trunk movements in the frontal, sagittal, and transverse planes during the whole gait cycle as the mean of three trials of the leg with lowest GDI at the CH and FU in the AMC groups is shown in Fig. 3.

There were no differences in time and distance parameters in any group between the left and right sides in cadence, walking speed, step length, and step width. Table 3 shows the average of time and distance parameters in AMC1, AMC2, and AMC3 at CH and FU.

**Table 3:** Cadence, walking speed, step length, and step width as averaged of the left and right sides in AMC1, AMC2 and AMC3 at gait analysis in childhood (CH) and gait analysis at follow-up (FU). “N” indicates non-dimensionalized. Time and distance parameters expressed as units are shown in Appendix B

	AMC1 (*n* = 3)			AMC2 (*n* = 5)			AMC3 (*n* = 4)		
Median [min, max]	CH	FU	*p*	CH-	FU	*p*	CH	FU	*p*
N Cadence	0.39 [0.35, 0.43]	0.40 [0.28, 0.43]	1.000	0.56 [0.45, 0.63]	0.54 [0.45, 0.59]	0.500	0.57 [0.55, 0.59]	0.58 [0.58, 0.62]	0.273
N Walking speed	0.29 [0.25, 0.29]	0.27 [0.19, 0.29]	1.000	0.38 [0.33, 0.47]	0.43 [0.29, 0.46]	0.893	0.44 [0.43, 0.46]	0.44 [0.42, 0.47]	0.715
N Step length	0.71 [0.66, 0.73]	0.68 [0.64, 0.72]	0.593	0.73 [0.66, 0.74]	0.76 [0.62, 0.84]	0.345	0.76 [0.73, 0.82]	0.76 [0.70, 0.79]	0.715
N Step width	0.23 [0.22, 0.31]	0.29 [0.25, 0.36]	0.109	0.21 [0.12, 0.25]	0.15 [0.14, 0.21]	0.138	0.16 [0.12, 0.18]	0.13 [0.11, 0.15]	0.273

### Satisfaction with orthoses at FU

**Fig. 4:**
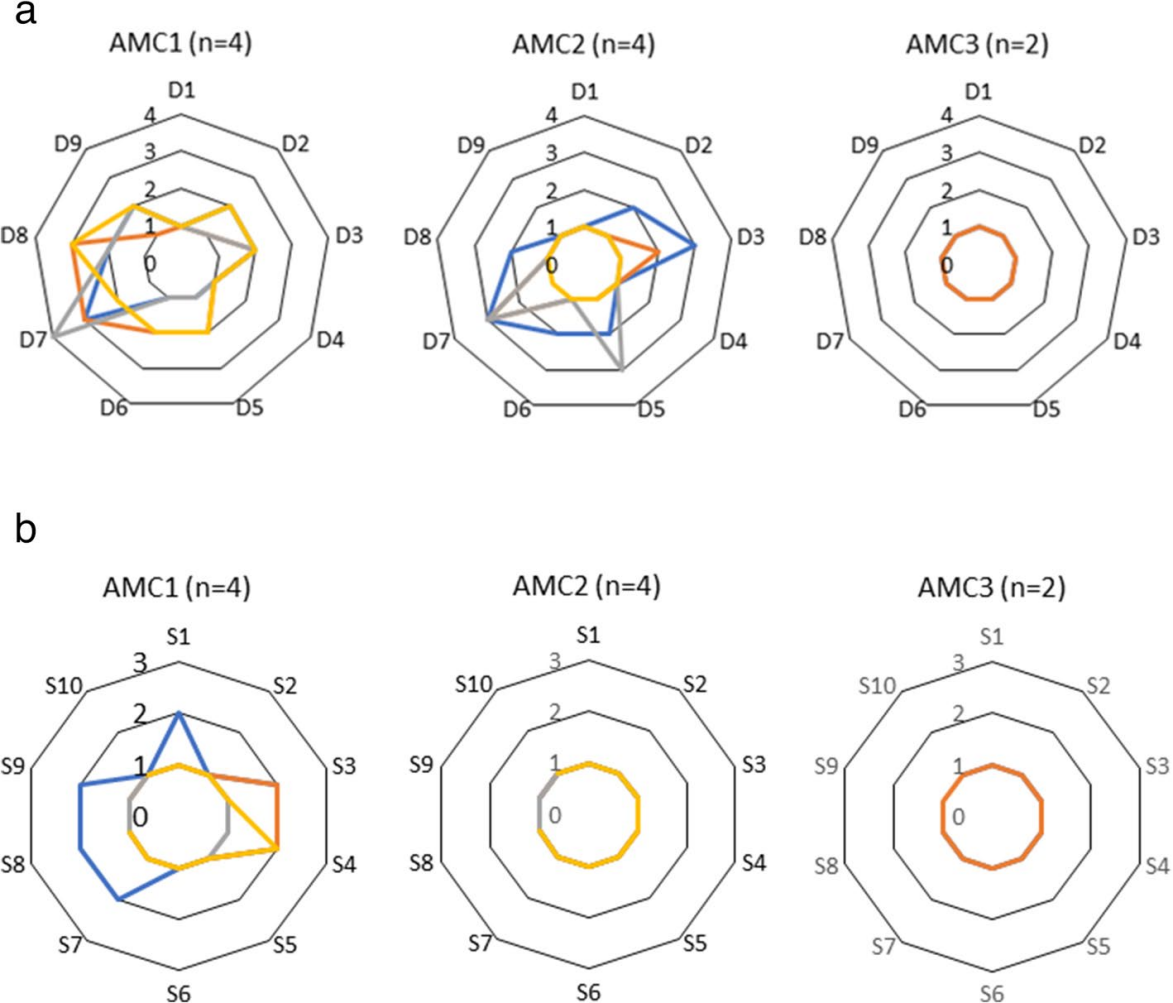
**a** Illustration of the single items of each participant according to AMC group in terms of client satisfaction with the device (CSD). D1: My orthosis fits well; D2: The weight of my orthosis is manageable; D3: My orthosis is comfortable throughout the day, D4:It is easy to put on my orthosis; D5: My orthosis looks good; D6: My orthosis is durable; D7: My clothes are free from wear and tear from my orthosis; D8: My skin is free from abrasions and irritations; and D9: My orthosis is pain free to wear. D: Device, Response scale for CSD: 1 = strongly agree, 2 = agree, 3 = disagree, 4 = strongly disagree. **b** Client satisfaction with the service (CSS). S1: I received an appointment with an orthotist within a reasonable amount of time; S2: I was shown the proper level of courtesy and respect by the staff; S3: I waited a reasonable amount of time to be seen; S4: Clinic staff fully informed me about equipment choices; S5: The orthotist gave me the opportunity to express my concerns regarding my equipment; S6: The orthotist was responsive to my concerns and questions; S7: I am satisfied with the training I received in the use and maintenance of my orthosis; S8: The orthotist discussed problems I might encounter with my equipment; S9: The staff coordinated their services with my therapists and doctors; and S10: I was a partner in decision making with clinic staff regarding my care and equipment. S: Survey, Response scale for CSS: 1 = strongly agree, 2 = agree, 3 = disagree

Ten participants who used orthoses answered the Swedish version of OPUS. The 9 items of CSD had a total score of median 68.7 [54.6, 100] and the 10 items of CSS had a total score of median 79.55 [64.3, 100]. There were no differences between the AMC groups in total scores of CSD, with AMC1, AMC2, and AMC3 having total scores of median 63.9 [54.6, 65.4], 100 [59.7, 100], and 100 [100, 100] (*p* = 0.076), nor in CSS, with total scores of 77.55 [64.3, 100], 89.0 [74.0, 100], 89.0 [78.0, 100] (*p* = 0.662), respectively. Figure 4 a-b illustrates the responses on the single items of each participant according to AMC group for the CSD and CSS.

### Report of a participant with gait analysis only at FU

The one participant that had performed a GA at follow-up but not in childhood was a male, 26 years of age, with AMC subtype pterygium with upper limb involvement in fingers and wrists. He had household ambulation, had used non-articulated KAFOs with hinged ankle joints since childhood, and was able to use a walker. At the current 3D examination, he preferred to walk without support. His orthoses had a weight of 3.5 kilogrammes, reporting to use them 7 days/week, > 10 h/day. He had bilateral hip flexion contractures of 45° bilaterally, knee flexion contractures of 75° and 80° on the left and right side, respectively, and no plantar flexor contractures. The muscle strength on left and right side was grade 4 and 5 in hip extension, grade 4 bilaterally in hip abduction, grade 0 and 3 in knee extension and grade 4 bilaterally in plantarflexion. A bilateral distal femoral osteotomy had been performed at 4 years of age and surgical scoliosis correction at 6 years of age. The calculated time and distance parameters from the three gait trials of the left and right sides were a walking cadence median [min, max] of 114 [109, 120] steps/minute, a walking speed of 0.76 [0.70, 0.82] metres/second, a step length of 0.40 [0.38, 0.43] metres and a step width of 0.10 [0.07, 0.12] metres.

## Discussion

In agreement with our hypothesis, the GDI remained consistent in AMC1 and AMC3 between the child and adult gait analyses. In AMC2, we found a decrease between the child and adult gait examinations as calculated from the leg with the lowest GDI with a median of approximately 11 scores. The gait analyses in childhood and adulthood were 6 to 12 years apart, making the choice of a multivariate measure of gait deviations such as the GDI [20] seem plausible. From calculating the left and right sides separately, the GDI score differed between CH and FU in both legs in AMC2. For further presentation, the leg with the lowest GDI score was selected. The GDI values found in this study could be viewed in relation to the results of control groups from the same gait laboratory, which consisted of 37 children with a GDI median [min, max] of 98.5 [95.1, 106.0] [5]. To simplify the kinematic data presentation only data from the control group of children from the gait laboratory were included in the figures. To calculate statistics for the kinematic variables, it was considered that the groups of AMC1, AMC2 and AMC3 were too small.

According to the original developers of the GDI concept [20], the angles in the sagittal, frontal, and transverse planes for the pelvis and the hip were incorporated in the GDI whereas only the angle in the sagittal plane was used for the knee. This limitation for the knee was due to coronal plane being prone to crosstalk from poor knee axis alignment. The ankle in the sagittal plane was considered most clinically utile and foot progression was selected as the most commonly used transverse plane for the measure of foot orientation [20]. Because the scope of presenting a GDI score is not to present values of single kinematic features, the measurements of these specific angles are not presented in this study.

GDI differed between CH and FU in AMC2 whereas no differences were found in AMC1 or AMC3. At FU none of the participants had a changed level of functional ambulation. Two participants in AMC2 had ceased using AFOs, of which one participant had changed to using insoles and one to using only shoes. The AMC subgroups were defined as in a previous study on gait dynamics in AMC [5]. Orthosis subgroups were used because they give an indication about which joints need to be stabilized. For example, the KAFO-L supports weak knee extensors, the KAFO-F prevents knee motion in the frontal and transverse planes and stabilizes the ankle, the AFO stabilizes the ankle, and the FO supports the plantar surface of the foot. Also, muscle strength measurements could indicate to what extent a muscle needs orthotic support. However, correct testing should be based on the whole range of motion of a joint, which is not always possible in joints with deformities [16]. Nevertheless, having information about muscle strength contributes to the understanding of a patient’s compensatory movements and gait pattern during walking. In the present study, 9 of the 12 participants were able to walk independently without orthoses whereas 3 required locked KAFOs to stand upright and walk. All participants were diagnosed with multiple congenital limb contractures and were considered free from involvement of central nervous system anomalies [7].

In terms of joint contractures, there were more improvements than deteriorations in joint motion and only two participants, one in AMC2 and one in AMC3, had gone through orthopaedic surgery in the interval between the CH and FU, and thus these factors did not seem to influence significantly the GDI. Regarding time and distance parameters, no significant change was found between CH and FU in any of the groups.

As expected, all groups maintained similar gait variations as they had in childhood, but there were deviations in all three biomechanical planes in all AMC groups, and the deviations were more apparent in AMC1 than in AMC2 or AMC3. As can be seen in the the figures that illustrate the kinematic parameters included in the GDI, the main attributes that were recognized in AMC1 were maintained knee extension angles followed by locked orthosis joints with increased dorsiflexion and hip flexion for alignment of the body segments in the sagittal plane. In AMC2 the main attributes were the absence of the third foot rocker due to the restricted dorsiflexion movement in the orthoses but with close to normal knee flexion–extension movement. In AMC3 the main attributes were the somewhat decreased dorsiflexion in stance but normal plantarflexion at the end of the stand phase, which was possible due to wearing insoles or shoes. The change from AFOs to insoles or shoes in the two participants in AMC2 did not seem to influence the ankle kinematics. In all groups, increased anterior pelvic tilt was observed at FU. In AMC3, there was an improvement with less anterior pelvic tilt and less hip flexion although not achieving neutral hip extension movement possibly due to extensive hip flexion contractures in one participant. In AMC1 and AMC2, anterior pelvic tilt had increased with increased hip flexion in AMC2, and with increased forward trunk tilt in AMC1. One participant in AMC1 reported that he had back pain almost every day, which he also stated during the gait analysis examination. It is likely that the back pain may have affected this participants’ posture when walking. Because trunk movements are not part of the GDI, these kinematic values were calculated extra. As observed from Fig. 3, AMC2 and AMC3 showed small differences in trunk movements between CH and FU. The characteristic increased trunk movements in the frontal plane as well as in the transverse plane of AMC1 were confirmed, as reported previously [5]. Taken together, although a detailed kinematic analysis has not been reported in this study, the gait patterns as summarized in the GDI mirror the movements that were used by persons with AMC.

Even if there were no differences in single CSD-items concerning satisfaction of the device (i.e., the orthosis), in AMC1 two participants disagreed with and one strongly disagreed with, “My clothes are free from wear and tear from my orthosis,” and two participants disagreed with, “My skin is free from abrasions and irritations.” In AMC2 one participant disagreed with, “My orthosis is comfortable throughout the day,” one disagreed with, “My orthosis looks good,” and three participants disagreed with, “My clothes are free from wear and tear from my orthosis.” Negative experiences with damage to clothing caused by Velcro straps or the mechanical knee joint in a KAFO was reported in adults with MMC [14]. Because similar orthosis designs and materials are chosen for persons with AMC and with MMC, these circumstances are likely to be the same for both groups. In contrast to persons with MMC, who have reduced tactile sensation that make them vulnerable for skin breakdown [25], persons with AMC are reported to have normal tactile sensation [26] that would possibly make them more sensitive to pressure from orthosis material. However, none of the eight participants disagreed with, “My orthosis is pain free to wear.” In AMC3, two participants agreed with all nine items, which may be plausible because insoles are likely to be less interfering with clothes and skin. It may seem remarkable that none in the AMC1 group disagreed with, “The weight of my orthosis is manageable,” because the weight of their KAFOs with locked knee joint was approximately twice the weights of the orthoses in AMC2. None of the single CSS items differed between the groups, nor did any of the participants disagree with the 10 items concerning service.

Because AMC is defined a rare condition [1], we were eager to collect as much information as possible from our population, and thus the gait in one adult who had performed the gait analysis only as an adult is also presented. Reporting of a participant who had not performed a gait analysis at childhood seemed important because it could add to the knowledge for early prescription of orthoses. In this case, a 26-year-old participant still walked with non-articulated KAFOs despite extensive hip and knee contractures, although with hinged AFOs. Concerning this participant’s time and distance parameters, he reached a cadence similar to AMC2, a walking speed similar to AMC1, a step length shorter than all groups, and a step width similar to AMC3. As formulated in the medical chart from the latest appointment with an orthopaedic surgeon, the desire for new gait orthoses suggests that ambulation is important in this participant’s life. It is likely that the available hip and plantarflexion muscle strength contributed to propel the body forwards and to generate biomechanical power in stance, which was sufficient for walking short distances indoors. Because the orthoses and shoes were adjusted with corresponding heel heights to achieve the best possible postural alignment given the knee and hip contractures, a valid marker placement could not be performed. Among the other 12 participants who had orthoses since childhood, all had continued wearing them, except one participant who had changed from AFOs to shoes. As stated above, all orthoses were fabricated by the same orthotic provider.

This study has several limitations. It should be mentioned that the follow-up was performed already during 2018–2019 that could mean that new orthosis models have been developed in the meantime. Even if this would be theoretically possible, the same orthosis provider still maintains contact with all participants certifying that orthosis design has not been changed in any of the participants. Using statistics in small sizes of study groups as in the present study can be questioned, and the heterogeneity among the participants probably influenced the results. The non-significant results between the AMC groups in the GDI score strengthen this assumption. The small study size is difficult to avoid because the disability is rare and thus the population in each geographical area is limited. From those two persons who denied participating in the gait analysis assessment, there is information that they are still ambulators using the same orthoses at the time of the present study as in childhood. In addition, when placing retroreflective markers to anatomical landmarks in the 3D gait analysis, the marker placement must be done with great accuracy to avoid measurement errors [27]. This concern is greater when placing markers on orthoses as applied in the present study. Moreover, because the initial gait assessment was performed in childhood, the body stature of the participants may have changed during the intervening years before FU. These challenges were hopefully overcome by reporting an overall gait index for the limbs with illustrations of the gait pattern corresponding to the summarized kinematic features.

## Conclusions

In a comparison of gait between childhood and adulthood, no differences were found in time and distance parameters that were normalized to leg length. In AMC2, remaining side differences and a slightly lower GDI score was seen at the follow-up than in childhood, which cannot be attributed to increased joint contractures, poorer muscle strength or orthopaedic surgery during the follow-up period. AMC1 showed increased pelvic anterior tilt with associated forward trunk tilt that may have been related to back pain in one participant. None of the participants had changed their functional walking ability as community and household ambulation. Two participants had reduced orthotic use from AFOs to shoes or insoles while all others had retained their orthotic supply from childhood. Neither satisfaction with the orthoses nor with the service at the CPO-clinic differed between the groups. This study reflects that gait is maintained in ambulating persons with AMC who were offered an orthosis program that has been available from childhood into adulthood.

## Supplementary Information

Supplementary Material 1.

Supplementary Material 2.

## Data Availability

No datasets were generated or analysed during the current study.

## Abbreviations

CH: Childhood
FU: Follow-up
GDI: Gait deviation index
AMC: Arthrogryposis multiplex congenita
AMC1: Arthrogryposis class 1
AMC2: Arthrogryposis class 2
AMC3: Arthrogryposis class 3
TD: Typically developing subjects
Ca: Community ambulation
Ha: Household ambulation
FO: Foot orthosis
AFO: Ankle–foot orthosis
AFO-S: Ankle–foot orthosis, solid
AFO-H: Ankle–foot orthosis, hinged
AFO-C: Ankle–foot orthosis, carbon fibre spring
KAFO: Knee-ankle–foot orthosis
KAFO-F: Knee-ankle–foot orthosis, free articulated
KAFO-L: Knee-ankle–foot orthosis, locked
OPUS: Orthotics and Prosthetics Users’ Survey
CSD: Client satisfaction with the device
CSS: Client satisfaction with services
