# Supplementary material for: Maintained gait in persons with arthrogryposis from childhood to adulthood
Source: BMC Musculoskelet Disord. 2025 Feb 12;26:141. doi: 10.1186/s12891-025-08366-9 (PMC11817894; doi:10.1186/s12891-025-08366-9)
Supplement: Supplementary file 1 — Supplementary Material 1. [file 12891_2025_8366_MOESM1_ESM.pdf]

Appendix A

Orthosis types and material used in the orthoses in the AMC groups at gait analysis in childhood (CH) and at follow-up (FU).

Subject	Orthosis type at CH/FU	CH	FU
**AMC1 (n=3)**			
1	KAFO-KL-C/KAFO-KL-C	Knee-ankle-foot orthosis with lockable knee joint:<br>*Thigh:* Composite<br>*Knee*: medio-lateral metal hinges with anterior lock mechanism<br>*Shank*: composite<br>*Ankle*: carbon fibre spring<br>*Foot*: composite merged with thermoplastic from the metatarsal heads 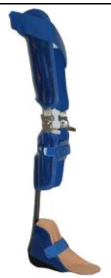	Unchanged orthosis type 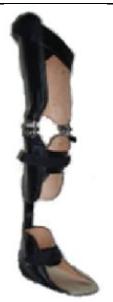
2	KAFO-KL/ KAFO-KL	Knee-ankle-foot orthosis with lockable knee joint:<br>*Thigh*: Composite<br>*Knee*: medio-lateral metal hinges with posterior lock mechanism<br>*Shank*: composite<br>*Ankle*: overlap thermoplastic/composite<br>*Foot*: thermoplastic 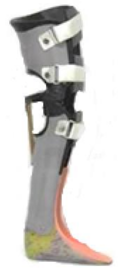	Unchanged orthosis type 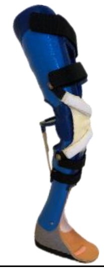
3	KAFO-KL-C/ KAFO-KL-C	Knee-ankle-foot orthosis with lockable knee joint:<br>*Thigh*: Composite<br>*Knee*: medio-lateral metal hinges with anterior knee lock mechanism<br>*Shank*: composite<br>*Ankle*: carbon fibre spring 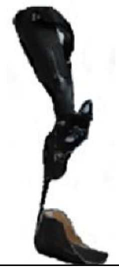	Unchanged orthosis type 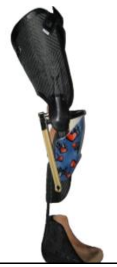

		Foot: composite merged with thermoplastic from the metatarsal heads	
**AMC2 (n=5)**			
4	AFO-S/AFO-C	AFO-S (AFO Solid):<br>*Shank*: thermoplastic<br>*Ankle*: unarticulated thermoplastic<br>*Foot*: thermoplastic 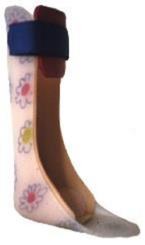	AFO-C (AFO with carbon fibre spring):<br>*Shank*: composite<br>*Ankle*: carbon fibre spring special ordered<br>*Foot*: composite merged with thermoplastic from the metatarsal heads 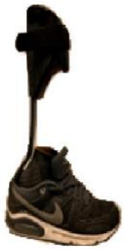
5	AFO-C/AFO-C	AFO-C (AFO with carbon fibre spring):<br>*Shank*: composite<br>*Ankle*: carbon fibre spring<br>*Foot*: composite merged with thermoplastic from the metatarsal heads 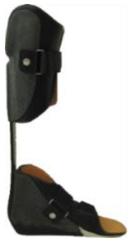	Unchanged orthosis type 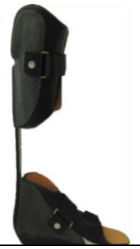
6	KAFO-F-C L & AFO-C R/ KAFO-F-C L & AFO-C R	KAFO-F-C (Knee-ankle-foot orthosis with free articulated knee joint):<br>*Thigh: composite*<br>*Knee*: monolateral metal hinge<br>*Shank*: composite<br>*Ankle*: Carbon fibre spring<br>*Foot*: composite merged with thermoplastic from the metatarsal heads<br>AFO-C (AFO with carbon fibre spring):<br>*Shank*: composite<br>*Ankle*: carbon fibre spring<br>*Foot*: composite merged with thermoplastic from the metatarsal heads. 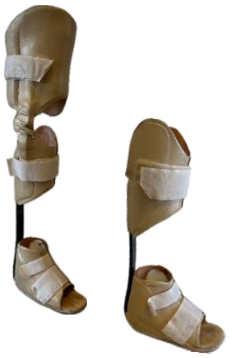	Unchanged orthosis types 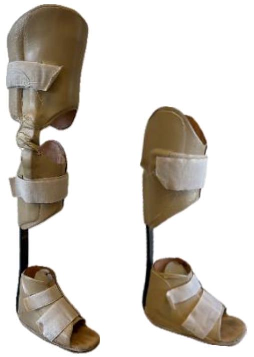

7	AFO-H/FO-insole	AFO-H (AFO-Hinged):<br>*Shank*: composite<br>*Ankle*: mediolateral metal hinges<br>*Foot*: composite merged with thermoplastic from the metatarsal heads. 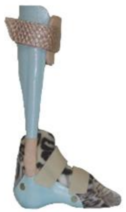	FO-insole (FO): Custom fabricated insoles EVA (ethylene vinyl acetate) 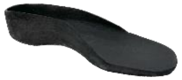
8	AFO-S L/Shoe	AFO-S (AFO Solid):<br>*Shank*: thermoplastic<br>*Ankle*: unarticulated thermoplastic<br>*Foot*: thermoplastic 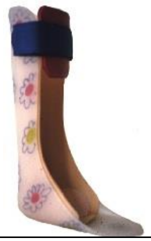	Shoe
**AMC3 (n=4)**			
9	FO-insole/FO-insole	FO-insole (FO): Custom fabricated insoles EVA (ethylene vinyl acetate) 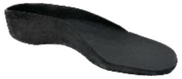	Unchanged orthosis type 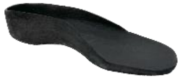
10	AFO-C/ FO-insole	FO-insole (FO): Custom fabricated insoles EVA (ethylene vinyl acetate) 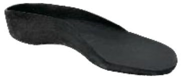	FO-insole (FO): Custom fabricated insoles EVA (ethylene vinyl acetate) 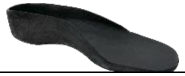
11	Shoe/Shoe	Shoe	Shoe
12	Shoe/ Shoe	Shoe	Shoe
