# Supplementary material for: Maintained gait in persons with arthrogryposis from childhood to adulthood
Source: BMC Musculoskelet Disord. 2025 Feb 12;26:141. doi: 10.1186/s12891-025-08366-9 (PMC11817894; doi:10.1186/s12891-025-08366-9)
Supplement: Supplementary file 2 — Supplementary Material 2. [file 12891_2025_8366_MOESM2_ESM.pdf]

Appendix B.

Cadence, walking speed, step length, and step width as averaged of the left and right sides in AMC1, AMC2 and AMC3 at gait analysis in childhood (CH) and gait analysis at follow-up (FU).

	AMC1 (n=3)		AMC2 (n=5)		AMC3 (n=4)	
Median [min, max]	CH	FU	CH	FU	CH	FU
Cadence (steps/min)	86<br>[78, 88]	80<br>[59, 88]	116<br>[92, 136]	111<br>[97, 118]	130<br>[111, 136]	120<br>[114, 135]
Walking speed (m/sec)	0.78<br>[0.68, 0.86]	0.81<br>[0.54, 0.87]	1.07<br>[0.96, 1.32]	1.23<br>[0.81, 1.36]	1.18<br>[1.11, 1.32]	1.26<br>[1.19, 1.42]
Step length (m)	0.54<br>[0.53, 0.58]	0.55<br>[0.55, 0.64]	0.58<br>[0.55, 0.62]	0.65<br>[0.50, 0.72]	0.54<br>[0.48, 0.71]	0.63<br>[0.54, 0.72]
Step width (m)	0.20<br>[0.16, 0.24]	0.25<br>[0.21, 0.32]	0.16<br>[0.10, 0.21]	0.13<br>[0.12, 0.18]	0.11<br>[0.09, 0.17]	0.12<br>[0.09, 0.14]
